# Calprotectin (S100A8/9) as serum biomarker for clinical response in proof-of-concept trials in axial and peripheral spondyloarthritis

**DOI:** 10.1186/s13075-014-0413-4

**Published:** 2014-08-19

**Authors:** Maureen C Turina, Nataliya Yeremenko, Jacqueline E Paramarta, Leen De Rycke, Dominique Baeten

**Affiliations:** Department of Clinical Immunology and Rheumatology, Academic Medical Center/University of Amsterdam, Amsterdam, The Netherlands; Laboratory of Experimental Immunology, Academic Medical Center/University of Amsterdam, Amsterdam, The Netherlands; Department of Rheumatology and Clinical Immunology, University Medical Center Utrecht, Utrecht, The Netherlands

## Abstract

**Introduction:**

Biomarkers complementing clinical evaluations may help to reduce the length and size of proof-of-concept (PoC) trials aimed to obtain quick “go/no go” decisions in the clinical development of new treatments. We aimed to identify and validate serum biomarkers with a high sensitivity to change upon effective treatment in spondyloarthritis (SpA) PoC trials.

**Methods:**

The candidate biomarkers high-sensitivity C-reactive protein (hs-CRP), interleukin-6 (IL-6), pentraxin-3 (PTX-3), alpha-2-macroglobulin (alpha-2-MG), matrix metalloproteinase-3 (MMP-3), calprotectin, and vascular endothelial growth factor (VEGF) were determined by enzyme-linked immunosorbent assay (ELISA) in healthy controls (*n* = 20) and SpA patients before and after 2 weeks of infliximab (*n* = 18) or placebo (*n* = 19) treatment in cohort 1. Clinical outcome was evaluated at week 12. Results were validated in ankylosing spondylitis (AS) with infliximab (cohort 2, *n* = 21) and peripheral SpA with etanercept (cohort 3, *n* = 20).

**Results:**

Serum levels of calprotectin, hs-CRP, PTX-3, VEGF (all *P* < 0.001) and MMP-3 (*P* = 0.062), but not IL-6 and alpha-2-MG, were increased in SpA versus healthy controls. Treatment with infliximab, but not placebo, significantly decreased calprotectin (*P* < 0.001) and hs-CRP (*P* < 0.001) levels, with a similar trend for MMP-3 (*P* = 0.063). The standardized response mean (SRM), which reflects the ability to detect changes over time, was high for calprotectin (−1.26), good for hs-CRP (−0.96) and moderate for MMP-3 (−0.52). Calprotectin and hs-CRP, but not MMP-3, were good biomarkers for treatment response in axial and peripheral SpA as evaluated and confirmed in cohort 2 and 3 respectively.

**Conclusions:**

Calprotectin and hs-CRP are good serum biomarkers with high sensitivity to change upon effective treatment at the group level in small-scale, short term PoC trials in SpA.

## Introduction

The use of tumor necrosis factor (TNF) blockers has dramatically improved the clinical outcome and quality of life of patients with spondyloarthritis (SpA). Originally implemented in ankylosing spondylitis (AS) and psoriatic arthritis (PsA), there is now increasing evidence that they are equally effective in other subtypes such as non-radiographic axial SpA and non-AS, non-PsA peripheral SpA [1-6]. Nevertheless, TNF blockade in SpA still has important limitations. First, contraindications and side effects, especially in patients with severe comorbidity, preclude the use of TNF blockers in a significant proportion of SpA patients. Second, TNF blockade fails to fully control signs and symptoms of disease in a significant proportion of patients, with only 15 to 20% reaching partial remission [1-6]. Third, the currently available evidence suggests that TNF blockade fails to halt new bone formation and ankylosis [7]. Finally, TNF blockade fails to induce long-lasting drug-free remission as disease relapses within a few months of treatment interruption in a large majority of patients [8,9].

These limitations indicate that there is still an important unmet medical need for alternative treatment options in SpA. However, the success of TNF blockade also raises important medical and ethical hurdles to perform large, long-term, placebo-controlled trials with novel drugs. Additional strategies to obtain quick go and no-go signals in small short-term proof-of-concept (PoC) trials before proceeding to large long-term clinical studies are thus required. Such tools may comprise biomarkers that reliably show high sensitivity to change upon clinical response at the group level. A biomarker is a ‘characteristic that can be objectively measured and evaluated as an indicator of a normal biologic process, a pathophysiologic process, or a pharmacologic response to a therapeutic intervention’ [10]. If sensitive and reproducible, changes in biomarker levels during treatment may thus help to substantiate a genuine effect on specific biological processes in PoC trials.

Several biomarkers have previously been directly or indirectly associated with treatment response in SpA. C-reactive protein (CRP) is known to be elevated in active disease and to decrease upon effective treatment in SpA, but is less useful than in rheumatoid arthritis (RA) as two thirds of the SpA patients have normal CRP levels [11-14]. IL-6 is the main driver of CRP; serum IL-6 levels are significantly increased in active SpA and decrease upon clinical response after TNF blockade [15,16]. Although CRP belongs to the short pentraxins, the pentraxin family also contains long pentraxins, including pentraxin-3 (PTX-3), which have been proposed as biomarkers of inflammation [17]. Alpha-2-macroglobulin (alpha-2-MG) is another CRP-independent acute-phase response protein that is used as serum biomarker [18].

Besides acute-phase response proteins, several potential biomarkers have been identified based on their involvement in the immunopathology of SpA. Matrix metalloproteinase-3 (MMP-3) levels are associated with peripheral arthritis, disease activity, progression of structural damage and response to TNF inhibition in SpA [19-21]. Calprotectin, a heterodimer consisting of S100A8 and S100A9 (previously called myeloid-related protein (MRP) 8 and 14, respectively), is expressed and secreted during monocyte infiltration into inflamed tissues, including macrophage infiltration in SpA synovitis [22-24]. Calprotectin serum levels are elevated in SpA, correlate well with disease activity and decrease upon TNF blockade [23]. Finally, SpA synovitis is characterized by pronounced hypervascularity [25,26] which is at least in part mediated by vascular endothelial growth factor (VEGF). VEGF serum levels are elevated and correlate well with inflammation and disease activity in SpA [27,28].

In this study, we aimed to systematically assess the value of these serum proteins as biomarkers of effective treatment in PoC trials with TNF blockers in SpA. More specifically, we aimed to identify which of these serum proteins can best discriminate between anti-TNF treatment and placebo as early as 2 weeks after start of treatment.

## Methods

### Patients and samples

Serum samples and clinical data for 20 healthy controls (HCs) and 78 SpA patients fulfilling the European Spondylarthropathy Study Group (ESSG) [29] criteria were retrieved from our arthritis biobank for this analysis according to the study protocol as approved by the Medical Ethics Committee of the Academic Medical Center/University of Amsterdam (2013_057). The SpA population consisted of three separate cohorts, where cohorts 2 and 3 are considered as independent validation cohorts. Cohort 1 consisted of 18 SpA patients with AS (n = 8), PsA (n = 8) and undifferentiated spondyloarthritis (USpA) (n = 2), who received infliximab (5 mg/kg intravenously) and 19 SpA patients with AS (n = 9), PsA (n = 9) and USpA (n = 1), who received placebo at weeks 0, 2 and 6 [1]. Cohort 2 consisted of 21 patients with AS according to the modified New York criteria, treated with infliximab (5 mg/kg intravenously) at weeks 0, 2 and 6 [30]. Cohort 3 consisted of 20 patients with peripheral SpA treated with etanercept (25 mg subcutaneously bi-weekly) [31]. None of the patients had been previously treated with a TNF blocker. Serum was obtained at baseline and week 2 for the first two cohorts, and at baseline and week 4 for cohort 3. The complete description of these cohorts and the response to treatment was reported previously [1,30,31].

### Serum biomarkers

We determined the serum levels of the following candidate biomarkers by ELISA as indicated by the manufacturers: high-sensitivity (hs)-CRP (Research & Diagnostic Systems, Inc. Minneapolis), IL-6 (Research & Diagnostics Systems, Inc.), PTX-3 (Research & Diagnostic Systems, Inc.), alpha-2-MG (Abcam, Cambridge, UK), MMP-3 (Biotrak, Amersham Pharmacia Biotech, Buckinghamshire, UK), VEGF (Research & Diagnostics Systems, Inc.), and Calprotectin (Hycult Biotech, the Netherlands). All assays were performed in duplicate.

### Statistical analysis

Data were represented as mean (standard error of the mean) and compared using the parametric *t*-test (paired where appropriate) using GraphPad Prism software version 5.01. A *P*-value below 0.05 was considered statistically significant. The standardized response mean (SRM) was calculated as the mean change in a parameter in a defined period of time divided by the SD of that change. An SRM below 0.5, between 0.5 and 0.8, and above 0.8 indicates a poor, moderate and good potential to detect changes over time, respectively [32]. Power calculations to attain 90% power with a significance level of 0.05 were performed using nQuery Advisor version 7.0 software.

## Results

### Baseline serum biomarkers in SpA versus HCs

**Figure 1:**
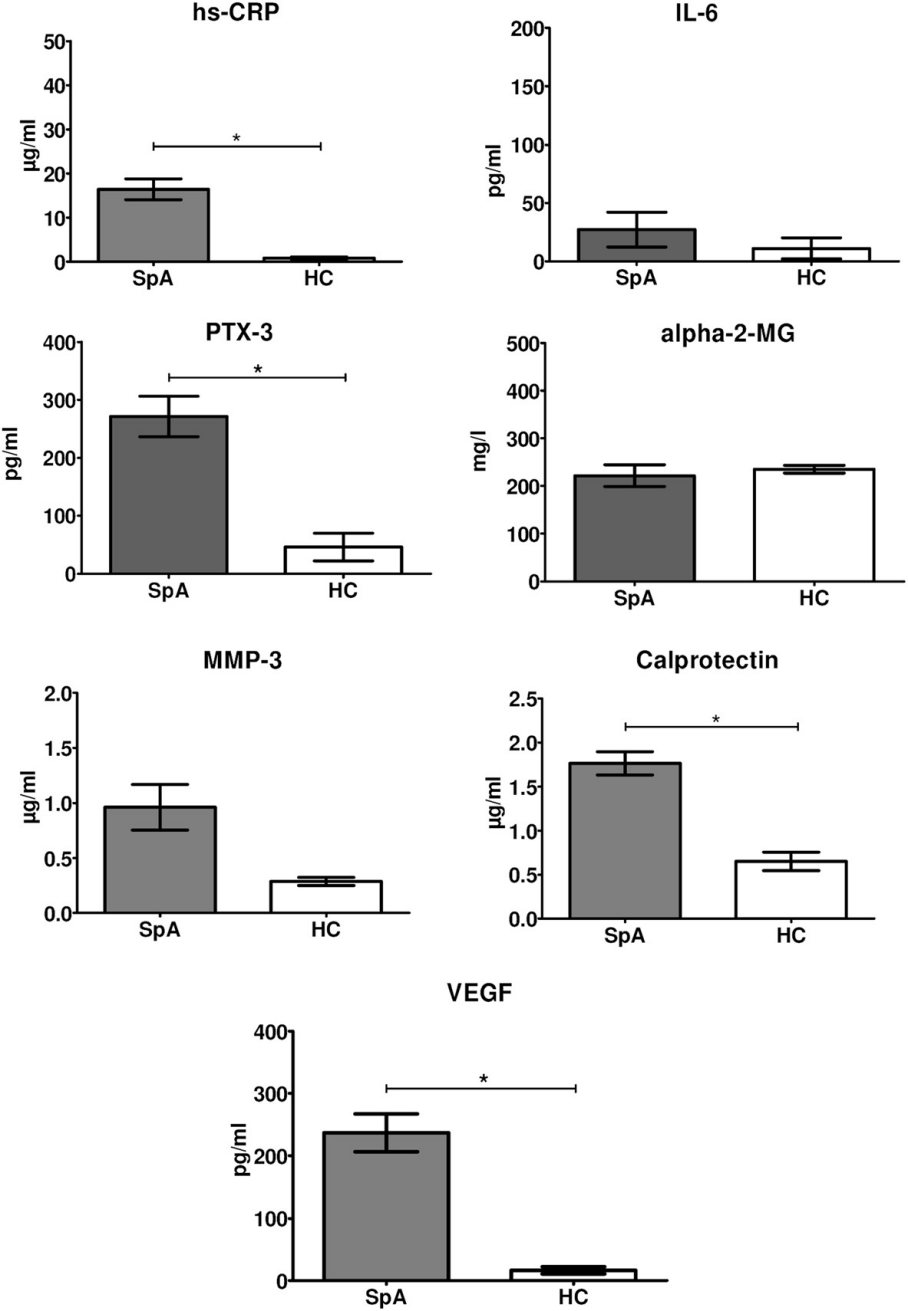
**Serum biomarker levels in active spondyloarthritis (SpA) (n = 37) versus healthy controls (HC) (n = 20).** Independent samples *t*-tests were performed. Data are represented as mean (standard error of the mean). *P <0.05. hs-CRP, high-sensitivity C-reactive protein; IL-6, interleukin-6; PTX-3, pentraxin-3; alpha-2-MG, alpha-2-macroglobulin; MMP-3, matrix metalloproteinase-3; VEGF, vascular endothelial growth factor.

We first assessed which serum biomarkers were increased in active SpA compared to HCs using the baseline samples of cohort 1 (Figure 1), assuming that serum biomarkers that are increased in active disease could potentially be modulated and normalized by effective treatment. We thus used this strategy to select biomarkers of interest; it was not the aim to identify markers with a high diagnostic discriminative capacity between SpA and HCs. The serum levels of hs-CRP (16.44 ± 2.36 μg/ml versus 0.84 ± 0.27 μg/ml; *P* <0.001), PTX-3 (271.4 ± 35.0 pg/ml versus 46.0 ± 23.6 pg/ml; *P* <0.001), calprotectin (1.76 ± 0.13 μg/ml versus 0.65 ± 0.10 μg/ml; *P* <0.001) and VEGF (237.0 ± 30.3 pg/ml versus 16.7 ± 5.7 pg/ml; *P* <0.001) were significantly higher in active SpA versus HCs, with a similar trend for MMP-3 (0.96 ± 0.21 μg/ml versus 0.29 ± 0.04 μg/ml; *P* = 0.062). In contrast, levels of alpha-2-MG and IL-6 were not significantly different between active SpA and HCs. Elevated serum levels, defined as greater than the mean + 2 SD of the serum levels of HCs, were observed in 86% of the SpA patients for calprotectin, 82% for VEGF, 81% for hs-CRP, 74% for MMP-3 and 59% for PTX-3.

### Short-term modulation of serum biomarkers by infliximab in SpA

**Figure 2:**
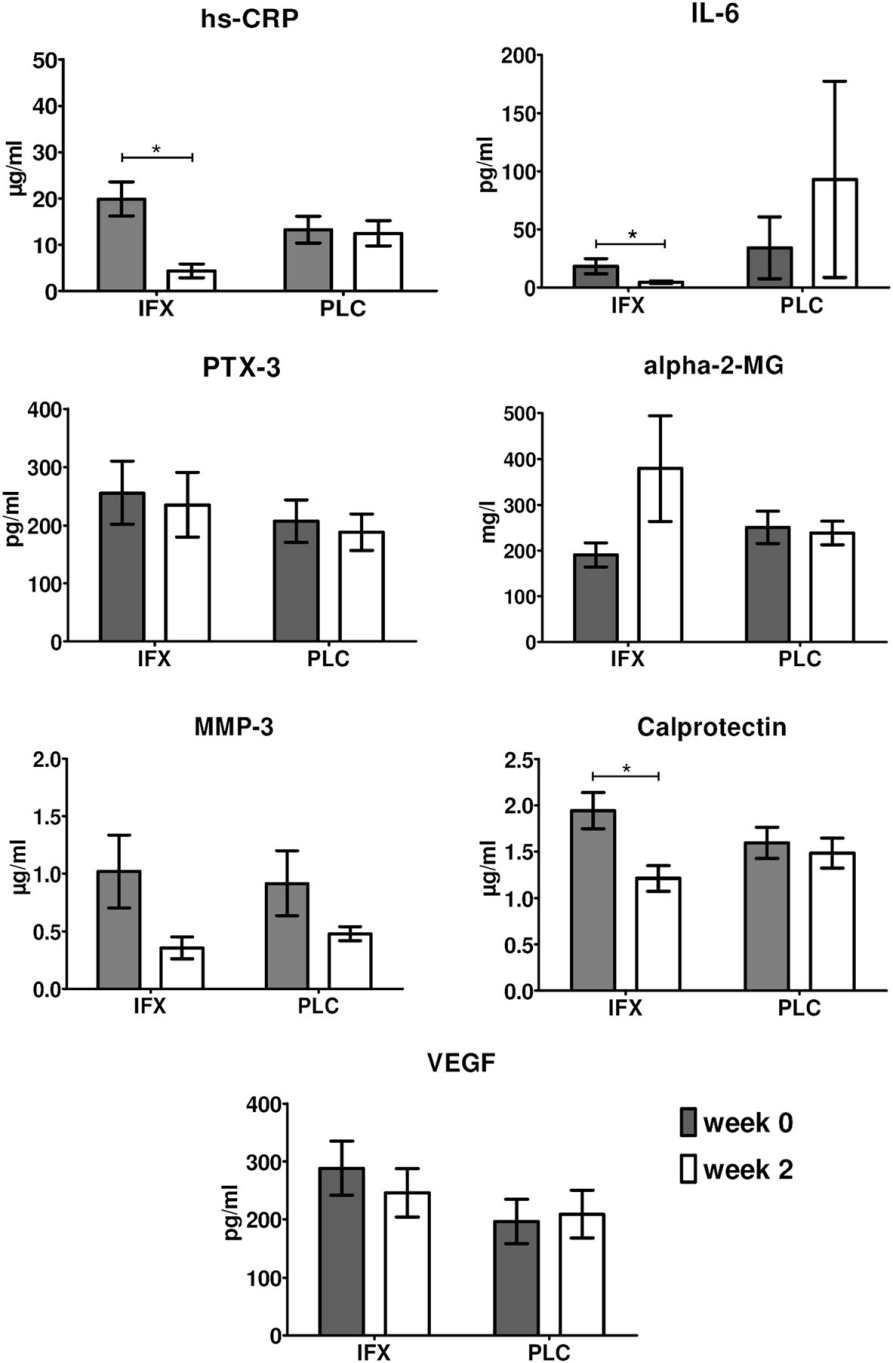
**Changes in serum biomarkers between baseline (week 0) and week 2 of infliximab (IFX) (n = 18) versus placebo (PLC) (n = 19) treatment in active spondyloarthritis** **(SpA).** Paired *t*-tests were performed. Data are represented as mean (standard error of the mean), *P <0.05. hs-CRP, high-sensitivity C-reactive protein; IL-6, interleukin-6; PTX-3, pentraxin-3; alpha-2-MG, alpha-2-macroglobulin; MMP-3, matrix metalloproteinase-3; VEGF, vascular endothelial growth factor.

We next assessed which of these biomarkers were significantly downregulated by infliximab but not placebo treatment as early as two weeks after initiation of therapy. We previously reported that both arms of this cohort were well-randomized for disease activity (as judged by parameters such as patient’s and physician’s global assessment of disease activity and Bath ankylosing spondylitis disease activity index (BASDAI), and that there was a significant decrease in these parameters during treatment with infliximab but not placebo [1]. Serum levels of the investigated biomarkers were similar at baseline between the infliximab and placebo arms of cohort 1 (Figure 2). In the placebo group, none of the serum biomarkers changed significantly between baseline and week 2 (Figure 2). In contrast, we observed a significant decrease in serum levels of hs-CRP (*P* <0.001), IL-6 (*P* = 0.047), and calprotectin (*P* <0.001) after two weeks of infliximab treatment, with a similar trend for MMP-3 (*P* = 0.063) (Figure 2). Levels of PTX-3, alpha-2-MG and VEGF were not significantly modulated by infliximab over this period.

### Potential of serum biomarkers to change sensitively upon active treatment in SpA

As the previous analyses identified calprotectin, hs-CRP, IL-6 and MMP-3 as the most promising biomarkers of treatment response, we next assessed their potential to change upon active treatment by using the SRM, which reflects the ability to detect changes over time [32]. The SRM in the infliximab group was −1.26 for calprotectin, −0.96 for hs-CRP, −0.56 for IL-6, and −0.52 for MMP-3. In comparison, the SRM in the placebo group was −0.20 for calprotectin, −0.13 for hs-CRP, −0.05 for IL-6, and 0.23 for MMP-3. Thus, the delta SRM (SRM of active treatment minus SRM of placebo) were −1.06 for calprotectin, −0.83 for hs-CRP, −0.51 for IL-6, and −0.75 for MMP-3. As calprotectin and hs-CRP correlated moderately (*r* = 0.648), we next aimed to confirm that an early change in calprotectin levels is superior to CRP as a biomarker of clinically effective treatment by performing a power analysis on the data of cohort 1. Demonstration of a significant difference between infliximab and placebo treatment using changes in calprotectin levels over two weeks as the outcome parameter with a power of 90% and a significance level of 0.05 would require 15 patients per group. A similar analysis with CRP would require 24 patients per group. Finally, we determined if a combination of changes in calprotectin and hs-CRP levels would be superior in treatment response compared to changes in calprotectin alone. Changes in calprotectin levels are independently associated to treatment response but when combined with changes to hs-CRP levels the association to treatment response was no longer significant.

### Serum biomarkers of active treatment in AS

**Figure 3:**
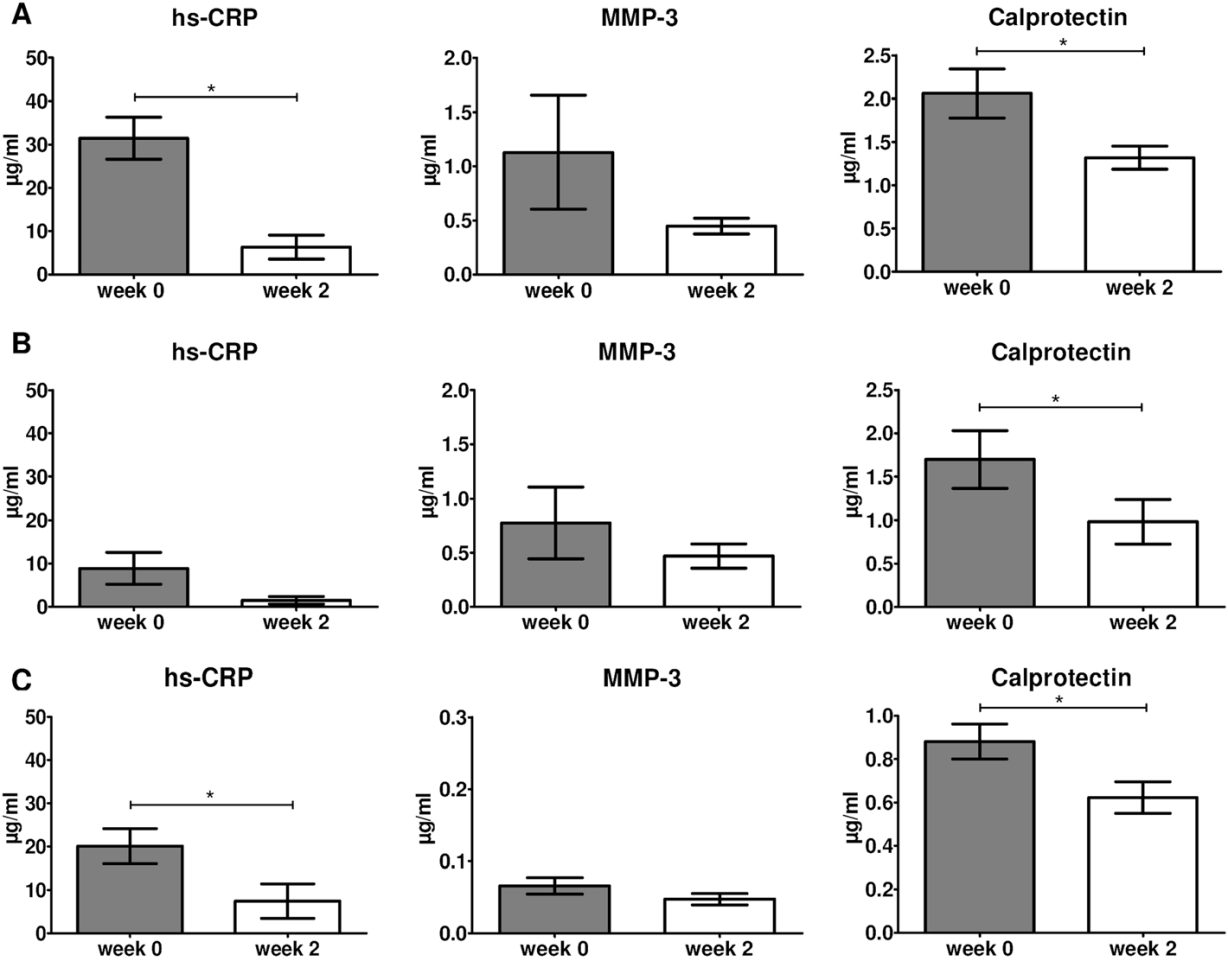
**Changes in serum biomarker levels between baseline (week 0) and week 2 of infliximab treatment in the ankylosing spondylitis (AS) patients (n = 8) (A) and psoriatic arthritis (PsA) patients (n = 8) (B) of cohort 1 as well as in the AS patients from cohort 2, who did not have peripheral disease (n = 21) (C).** Paired *t*-tests were performed. Data are represented as mean (standard error of the mean). *P <0.05. hs-CRP, high-sensitivity C-reactive protein; MMP-3, matrix metalloproteinase-3.

Cohort 1 consisted of different SpA subtypes, mainly AS and PsA. To ascertain that the biomarker findings are not biased by the SpA subtype and that the data can be extrapolated to more homogenous SpA study populations, we performed two additional sets of analysis. First, we reanalyzed the biomarker data of cohort 1 separately for AS (n = 8 in the infliximab group and n = 9 in the placebo group) and PsA (n = 8 in the infliximab group and n = 9 in the placebo group). In the AS group, only hs-CRP (*P* = 0.006) and calprotectin (*P* = 0.028) decreased significantly after two weeks of infliximab treatment (Figure 3A). Decreases in IL-6 and MMP-3 levels were not significant and none of the biomarkers changed in the placebo arm (data not shown). The SRM was −0.98 for calprotectin and −1.39 for hs-CRP. For PsA patients, only calprotectin (*P* = 0.003) decreased significantly after two weeks of infliximab treatment, with a similar but non-significant trend for hs-CRP (*P* = 0.071) (Figure 3B). None of the biomarkers changed in the placebo arm (data not shown). The SRM in PsA patients was −1.58 for calprotectin and −0.75 for hs-CRP.

Second, we performed a similar biomarker analysis in an independent cohort of patients with AS without peripheral arthritis or enthesitis (n = 20) receiving infliximab at weeks 0, 2 and 6 (cohort 2). As shown in Figure 3C, hs-CRP (*P* = 0.001) and calprotectin (*P* = 0.019) serum levels decreased significantly after two weeks of infliximab treatment. MMP-3 (*P* = 0.093) showed a similar but non-significant trend in this cohort. The SRM was −0.56 for calprotectin and −0.59 for hs-CRP. Taken together, these data indicate that changes in calprotectin and hs-CRP serum levels reflect well the response to active treatment with infliximab in AS.

### Serum biomarkers of treatment response in peripheral SpA

**Figure 4:**
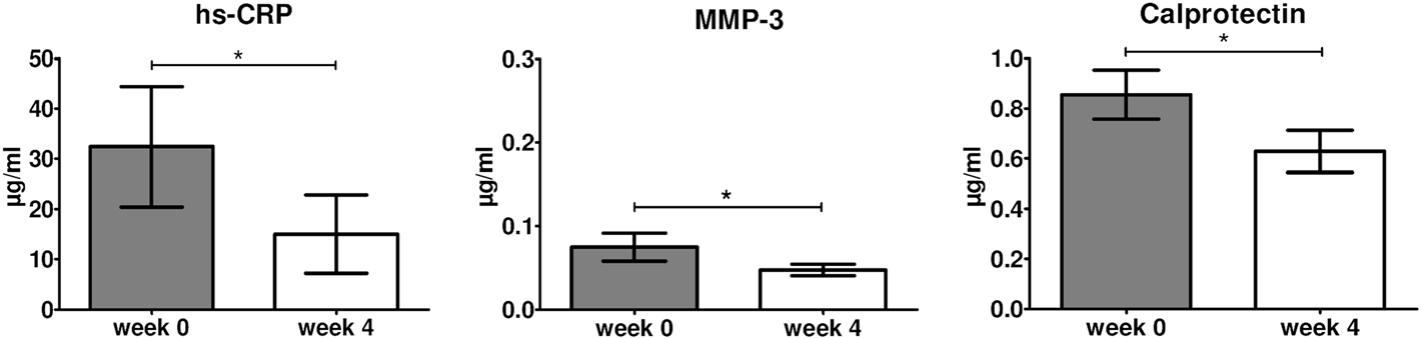
**Changes in serum biomarkers between baseline (week 0) and week 4 of etanercept treatment (n = 20) in peripheral spondyloarthritis (SpA).** Paired *t*-tests were performed. Data are represented as mean (standard error of the mean). *P <0.05. hs-CRP, high-sensitivitivity C-reactive protein; MMP-3, matrix metalloproteinase-3.

The previous analyses suggest that there may be some differences in serum biomarkers between AS and PsA. To confirm and extend these data and to ascertain that these biomarkers do not only reflect response to infliximab but also other TNF inhibitors, we analyzed changes in serum biomarkers between baseline and week 4 of etanercept treatment in patients with peripheral SpA (cohort 3). As shown in Figure 4, we again observed a significant decrease in serum levels of calprotectin (*P* = 0.032), hs-CRP (*P* = 0.035) and MMP-3 (*P* = 0.045). In this cohort, the SRM was −0.55 for calprotectin, −0.54 for hs-CRP, and −0.51 for MMP-3. Furthermore, changes in calprotectin levels were not influenced by the presence of psoriasis.

### Calprotectin as a biomarker in CRP-negative patients

Both calprotectin and CRP are not highly specific to SpA and are elevated in several diseases. In SpA, CRP levels were not elevated in two thirds of the patients. Furthermore, the correlation between CRP and calprotectin was modest in SpA (*r* = 0.634), suggesting that calprotectin may provide more or additional information in comparison with CRP. We analyzed whether calprotectin is of additional value by determining the sensitivity to change upon treatment effect in CRP-negative patients. We therefore stratified for CRP status (positive or negative), using the threshold for normal values from our local laboratory.

For cohort 1, the delta calprotectin levels were −0.30 ± 0.21 μg/ml in CRP-negative patients versus −0.90 ± 0.13 μg/ml in CRP-positive patients, with a corresponding SRM of −0.79 and −1.60 in CRP-negative and CRP-positive patients respectively. For cohort 2, the delta calprotectin levels were −0.19 ± 0.14 μg/ml in CRP-negative patients versus −0.35 ± 0.15 μg/ml in CRP-positive patients, with a corresponding SRM of −0.38 and −0.81 in CRP-negative and CRP-positive patients respectively. For cohort 3, the delta serum calprotectin levels were −0.10 ± 0.11 μg/ml in CRP-negative patients versus −0.30 ± 0.18 μg/ml in CRP-positive patients, with a corresponding SRM of −0.35 and −0.66 in CRP-negative and CRP-positive patients respectively.

## Discussion

Tissue inflammation is one of the major features of both axial and peripheral SpA but remains difficult to quantify and monitor in an objective and reliable way in clinical research. The most commonly used biomarkers for inflammation in SpA are magnetic resonance imaging (MRI) and CRP. MRI is able to visualize inflammatory lesions such as synovitis, enthesitis and bone marrow edema in axial and peripheral SpA and these lesions decrease or even disappear upon effective treatment [33,34]. Accordingly, MRI is now used as a measure of inflammation in clinical trials in AS and axial SpA. However, even in patients with high clinical disease activity MRI scores are often low, which makes it difficult to detect significant pre- and post-treatment changes in small PoC trials [35]. The second widely used measure of inflammation in SpA is CRP. CRP levels do correlate at the group level with clinical disease activity, decrease upon effective treatment and are included in the ankylosing spondylitis disease activity score (ASDAS) [36]. As for MRI, however, an important issue is that baseline serum levels of CRP are elevated only in a fraction of patients with active SpA, hs-CRP may therefore perform better than classical CRP measurements [13,37].

In the current study we explored the value of different candidate serum biomarkers of clinical response in SpA, thereby focusing specifically on small, short-term PoC trials. We aimed to identify serum proteins that are significantly modulated by effective treatment with TNF blockers but not by placebo, as early as 2 weeks after treatment initiation. In line with the previously discussed data, we found that hs-CRP is a useful biomarker of inflammation in this context as, despite not being elevated in all patients at baseline, it did rapidly and significantly decrease in both axial and peripheral SpA treated with either infliximab or etanercept. The high SRM and the absence of changes in the placebo-treated patients confirm its value as a biomarker of inflammation in PoC trials. Interestingly, serum levels of IL-6, the major driver of CRP, did not appear to be a reliable marker of treatment response in our study. Moreover, two recent clinical trials with IL-6-receptor blockers failed to demonstrate clinical efficacy in AS [38,39]. Taken together, these data suggest that, even though CRP may be used as biomarker, the IL-6-CRP axis is not a pivotal inflammatory pathway in SpA.

The major finding of the study is that serum levels of calprotectin appeared to be the most robust and reliable marker of treatment response in both axial and peripheral SpA, even outperforming hs-CRP in some analyses. For example, a power calculation in the infliximab cohort indicated that the use of calprotectin rather than hs-CRP as biomarker for inflammation would allow significant reduction in the size of the cohort. In contrast to CRP, there is also clear evidence that calprotectin directly reflects key inflammatory processes in SpA. Calprotectin is produced by myeloid cells and neutrophils and is secreted when these cells transmigrate through the endothelium to the inflamed tissues. Elevated levels of calprotectin as measured in synovial fluid in arthritis or in the feces in inflammatory bowel disease thus reflect local tissue infiltration by myeloid cells [40,41]. We and others have demonstrated that calprotectin is released locally in SpA synovitis, that the degree of synovial infiltration with myeloid cells and neutrophils reflect disease activity in peripheral SpA, and that effective treatment of peripheral SpA is associated with a rapid and robust decrease of calprotectin-positive cells in the inflamed synovium [22-24,42]. In contrast, much less is known about calprotectin expression in axial disease. Taken together, these data indicate that secretion of calprotectin may be a good reflection of local migration and activation of inflammatory innate immune cells in target tissues of SpA. Accordingly, a decrease in these calprotectin levels upon treatment is likely to reflect a genuine biological effect on chronic tissue inflammation.

Although we used only TNF blockers as effective drugs in the present study, we now have evidence that the biomarker value of calprotectin is not restricted to this sole mode of action. In a recent PoC study with the monoclonal anti-IL17A antibody, secukinumab, we demonstrated that both CRP and S100A8 and A9 levels decreased rapidly in the treated patients [35]. Supporting the concept that calprotectin may have additive value compared to CRP, changes between baseline and week 6 levels of S100A8 and A9, but not CRP, correlated with assessment of spondyloarthritis 20 (ASAS20) and ASAS40 responses at week 6. Moreover, baseline levels of S100A8 and S100A9 but not CRP had a high sensitivity to change upon clinical response to treatment in this trial.

The combination of calprotectin and hs-CRP as biomarkers for clinical response, does not give any additional value compared to calprotectin alone. In contrast to calprotectin and hs-CRP, the other candidate biomarkers did not perform that well in our studies. In line with previous reports [16,20,28,43], serum levels of markers such as VEGF and MMP-3 did decrease upon effective anti-inflammatory therapy but the treatment effects were either smaller or less consistent across different SpA subtypes and cohorts in comparison with calprotectin and hs-CRP. This may also be due to the specific setting of the present study where we aimed to detect very early changes (at week 2) in biomarker levels.

It is important to emphasize here that our data certainly do not imply that serum biomarkers such as calprotectin or hs-CRP should replace clinical outcome parameters. Indeed, clinical outcomes (including composite indices such as BASDAI and ASDAS) can also show significant and rapid changes in anti-TNF treated patients. However, the correlation between a serum biomarker such as calprotectin and clinical outcomes such as BASDAI is significant but weak (*r* = 0.325), indicating that both measurements do not cover exactly the same aspects of the disease. Clinical outcome parameters may also be more sensitive to placebo responses and thus combination of clinical outcome measures with serum biomarkers may help to obtain a consistent picture which can support go or no-go decisions in proof-of-concept trials. A second aspect to emphasize is that calprotectin, similar to CRP, is not exclusively elevated in SpA but also in many other inflammatory conditions [44]. Comorbidity may influence serum levels of calprotectin and CRP in SpA. Nevertheless, we demonstrated here that effective treatment still induces rapid, robust and consistent changes in these serum biomarkers, which are not observed during placebo treatment.

Our study has two important limitations. First, we used a candidate approach based on a limited set of potential biomarkers emerging from literature and/or insights into tissue inflammation in SpA. However, a more systematic approach using proteomics or gene expression arrays allowing identification of novel biomarkers, therefore, certainly remains warranted. For example, an unbiased microarray analysis of peripheral blood cells could identify a set of genes with expression that is closely correlated to disease activity in AS; one of these genes codes for LIGHT and serum levels of LIGHT have been confirmed to correlate with CRP and erythrocyte sedimentation rate (ESR) [45]. Second, we focused on the use of serum biomarkers in a very specific setting, namely the measurement of biological impact on inflammation in PoC trials. The set-up and size of the studies used in our analyses do not allow determination of whether changes in calprotectin and hs-CRP levels not only reflect treatment response at the group level but also allow discrimination at the individual level of responders from non-responders to TNF blockade. Moreover, it remains unknown whether serum biomarkers such as calprotectin may also reflect other important clinical features of SpA. As, for example, both CRP and VEGF serum levels correlate with osteoproliferation in axial SpA [46,47], it would be interesting to explore the value of calprotectin in similar settings. Finally, we did not explore the value of calprotectin as a biomarker of early disease, including non-radiographic axial SpA and patients presenting with early inflammatory back pain.

## Conclusions

In summary, good biomarkers are needed in the development of novel therapies in SpA to provide quick go and no-go signals for PoC trials. Our data indicate that calprotectin and CRP perform well as serum biomarkers, as these biomarkers have a high sensitivity to change upon clinical effective treatment and could be of use in the development of new treatments.

## Abbreviations

Alpha-2-MG: alpha-2-macroglobulin
AS: ankylosing spondylitis
ASAS: assessment of spondyloarthritis
ASDAS: ankylosing spondylitis disease activity score
BASDAI: Bath ankylosing spondylitis disease activity index
CRP: C-reactive protein
ELISA: enzyme-linked immunosorbent assay
ESR: erythrocyte sedimentation rate
ESSG: European Spondylarthropathy Study Group
HC: healthy control
hs-CRP: high-sensitivity C-reactive protein
IFX: infliximab
IL-6: interleukin-6
MMP-3: matrix metalloproteinase-3
MRI: magnetic resonance imaging
MRP8 and 14: myeloid-related protein 8 and 14
PLC: placebo
PoC: proof-of-concept
PsA: psoriatic arthritis
PTX-3: pentraxin-3
RA: rheumatoid arthritis
SpA: spondyloarthritis
SRM: standardized response mean
TNF: tumor necrosis factor
USpA: undifferentiated spondyloarthritis
VEGF: vascular endothelial growth factor
